# Shortening the Alzheimer’s disease assessment scale cognitive subscale

**DOI:** 10.1192/j.eurpsy.2024.14

**Published:** 2024-02-23

**Authors:** Stephen Z. Levine, Yair Goldberg, Anat Rotstein, Myrto Samara, Kazufumi Yoshida, Andrea Cipriani, Takeshi Iwatsubo, Stefan Leucht, Toshiaki A. Furukawa

**Affiliations:** 1 School of Public Health, University of Haifa, Haifa, Israel; 2 The Faculty of Data and Decision Science, Technion Israel Institute of Technology, Haifa, Israel; 3 Department of Gerontology, University of Haifa, Haifa, Israel; 4 Department of Psychiatry, Faculty of Medicine, University of Thessaly, Larissa, Greece; 5 Department of Health Promotion and Human Behavior, Graduate School of Medicine/School of Public Health, Kyoto University, Kyoto, Japan; 6 Department of Psychiatry, University of Oxford, Oxford, UK; 7 Oxford Health NHS Foundation Trust, Warneford Hospital, Oxford, UK; 8 Oxford Precision Psychiatry Lab, NIHR Oxford Health Biomedical Research Centre, Oxford, UK; 9 Department of Neuropathology, Graduate School of Medicine, The University of Tokyo, Bunkyo-ku, Tokyo, Japan; 10 Technical University of Munich, TUM School of Medicine and Health, Department of Psychiatry and Psychotherapy, München, Germany

**Keywords:** Alzheimer’s disease, assessment, clinical trials, cognition, item response theory, psychometric

## Abstract

**Background:**

A short yet reliable cognitive measure is needed that separates treatment and placebo for treatment trials for Alzheimer’s disease. Hence, we aimed to shorten the *Alzheimer*’*s Disease Assessment Scale Cognitive Subscale* (ADAS-Cog) and test its use as an efficacy measure.

**Methods:**

Secondary data analysis of participant-level data from five pivotal clinical trials of donepezil compared with placebo for Alzheimer’s disease (N = 2,198). Across all five trials, cognition was appraised using the original 11-item ADAS-Cog. Statistical analysis consisted of sample characterization, item response theory (IRT) to identify an ADAS-Cog short version, and mixed models for repeated-measures analysis to examine the effect sizes of ADAS-Cog change on the original and short versions in the placebo versus donepezil groups.

**Results:**

Based on IRT, a short ADAS-Cog was developed with seven items and two response options. The original and short ADAS-Cog correlated at baseline and at weeks 12 and 24 at 0.7. Effect sizes based on mixed modeling showed that the short and original ADAS-Cog separated placebo and donepezil comparably (ADAS-Cog original ES = 0.33, 95% CI = 0.29, 0.40, ADAS-Cog short ES = 0.25, 95% CI =0.23, 0.34).

**Conclusions:**

IRT identified a short ADAS-cog version that separated donepezil and placebo, suggesting its clinical potential for assessment and treatment monitoring.

## Introduction

Alzheimer’s disease is a progressive neurodegenerative disorder that cumulates in mortality on average 4–8 years after the diagnosis, characterized by impairments in the activities of daily functioning and cognitive decline [1]. Since cognitive impairment is a clinical hallmark of Alzheimer’s disease [1] suitable assessments are essential for treatment and research following onset [2]. The most widely used and researched cognitive impairment outcome in clinical trials of Alzheimer’s disease is the Alzheimer’s disease Assessment Scale Cognitive Subscale (ADAS-Cog) [3]. The ADAS-Cog is one of the two primary cognitive outcome measures required by the Food and Drug Administration for clinical drug trials for the treatment of Alzheimer’s disease in the United States [4]; however, it is quite long to administer (takes on average 30–35 min to complete).

Early evidence based on traditional psychometric approaches reported that the ADAS-Cog demonstrates acceptable levels of reliability and validity [1, 2]. Validity was supported based on evidence showing that the different aspects of cognition that constitute the ADAS-Cog are adequately correlated to form a single factor [3]. However, subsequent research did not replicate the single-factor solution and instead identified two- and three-factor solutions [4, 5] and queried the level of reliability of the ADAS-Cog [6]. Furthermore, some studies suggest that the ADAS-Cog is appropriate for use only in the moderate stages of cognitive impairment. Namely, the ADAS-Cog demonstrates severe floor (i.e., some items are too easy for patients) and ceiling (i.e., some items are too difficult for patients) effects [3, 7, 8]. Hence, contentions exist that the ADAS-Cog is inappropriate for mild and severe stage dementia [3, 7, 8]. In addition, the traditional psychometric approaches to examining the ADAS-Cog cannot examine treatment effects [3, 9, 10]. Hence, given these inconsistent findings, examination of the ADAS-Cog using advanced psychometric approaches is warranted.

To improve the ADAS-Cog, advanced psychometric approaches, such as item response theory (IRT), may be helpful [6]. Unlike traditional psychometric approaches, like factor analysis, IRT offers ADAS-Cog details at different cognitive impairment levels by item, information (i.e., reliability), and response option. It does so graphically and numerically. Estimates are available to map the ability of an item to discriminate underlying cognitive impairment levels. Also, it is possible to estimate the probability of progressing to a higher cognitive impairment response option rating or not. It is possible to identify which response options are likely, unlikely, and superfluous [11]. This feature of IRT is related to identifying items and response options that display ceiling or floor aspects on the ADAS-Cog. This seems of note to clinical trials where a given item may be used as a selection criterion, thereby impacting the response option ratings on the remaining items.

IRT has been implemented in studies to shorten psychiatric [9–11] and cognitive measures in dementia [12]. Studies that use IRT to examine the ADAS-Cog highlight that the measure is optimal within the moderate range of cognitive impairment only [13]. However, research has yet to identify an ADAS-Cog IRT-based shortened version that separates treatment and placebo to detect treatment effects.

We aimed to develop an ADAS-Cog short form (ADAS-Cog) using IRT based on individual-level participant clinical trial data and to examine whether it could separate treatment and placebo groups.

## Methods

### Participants

#### Study design

Data were accessed on pivotal individual-level participant data of randomized controlled double-blinded trials of donepezil conducted by Eisai Co. Ltd (see Table S1 published as supplementary material online attached to the electronic version of this paper at https://www.cambridge.org/core/journals/european-psychiatry). Data access was granted after the submission of an analytic plan. The data were analyzed on a secure Internet cloud-based platform (http://www.clinicalstudydatarequest.com). Trials were included in which participants with Alzheimer’s disease were assessed with the ADAS-Cog. Individual-level participant data were ascertained from five randomized clinical trials with similar follow-up intervals [14–18]. Institutional review boards approved each trial.

### Measures

*ADAS-Cog*: The ADAS-Cog is a neuropsychological index of cognitive impairment, indicating the severity of cognitive symptoms in Alzheimer’s disease [19]. This measure has been widely used in Alzheimer’s disease clinical trials [3] and has become as the gold standard for evaluating treatment efficacy [20]. It consists of 11 items to assess memory, language, and praxis functions [19]. The ADAS-Cog total score ranges from 0 to 70, with high scores indicating more severe cognitive impairment.

### Analytic plan

First, following the removal of individuals with missing baseline ADAS-Cog item level scores (Table 1), the analytic sample was characterized. Second, items and rating options were removed based on IRT to identify an ADAS-Cog short version. Third, the ADAS-Cog original and short versions were examined with mixed-effects models for repeated-measures analysis (MMRM).

**Table 1.** Sample characteristics

Study	N	Donepezil N (%)	Placebo N (%)	Female N (%)	Male N (%)	Age Mean (SD)
All trials	2198	1435 (65.29)	763 (34.71)	1344 (61.15)	854 (38.85)	72.42 (7.47)
Homma, Takeda (14)	268	136 (50.75)	132 (49.25)	179 (66.79)	89 (33.21)	70.51 (7.16)
Rogers and Friedhoff (15)	161	121 (75.16)	40 (24.84)	97 (60.25)	64 (39.75)	72.04 (7.45)
Rogers, Doody (16)	481	324 (67.36)	157 (32.64)	305 (63.41)	176 (36.59)	73.95 (7.56)
Rogers, Farlow (17)	473	311 (65.75)	162 (34.25)	293 (61.95)	180 (38.05)	73.48 (7.17)
Burns, Rossor (18)	815	543 (66.63)	272 (33.37)	470 (57.67)	345 (42.33)	71.62 (7.44)

### IRT of the ADAS-Cog at baseline

IRT assumes a single component underlies the data. Hence, principal components analysis was implemented to ascertain the number of components underlying the data. Next, the graded response model (GRM) [21], a form of IRT, was implemented in the ltm package in R [22]. The GRM model has been used to shorten measures previously [9–11, 23]. In IRT, item discrimination parameters (α) map the ability of an item to discriminate impairment levels. Discrimination parameter values for items are considered very low (between 0.01 and 0.24), low (0.25 and 0.64), moderate (0.65 and 1.34), high (1.35 and 1.69), and very high (over 1.7) [24]. Threshold parameters (βs) indicate the point at which there is a probability of endorsing a higher cognitive impairment rating than the previous rating option. If a threshold value exceeds 1.96, it suggests that ratings provide accurate information, and the converse applies to negative values.

Three graphs are used in IRT: item response category characteristic curves (a plot of the probability of endorsing a rating option by the level of underlying cognitive impairment), Item information curves (lines at similar information levels indicate overlapping, namely that the items assess similar information and so there exists a degree of item redundancy). Test information shows the reliability of the cognitive functioning assessment at different impairment levels.

### Mixed models to assess treatment effects

We examined change scores, marginal means, and effect sizes differences in the marginal mean with their associated bootstrapped confidence intervals between the donepezil and placebo groups using a three-level MMRM analysis with maximum likelihood estimation. The levels accounted for the data structure such that level 1 represented the visit, level 2 represented the individual, and level 3 represented the trial [25]. The covariates were age, sex, baseline ADAS-Cog score, and treatment group, and the outcome was the change score from baseline.

## Results

### Trial characteristics

After removing 12 participants owing to missing ADAS-Cog item responses, the five trials comprised 2,198 study participants. These formed the basis for the baseline IRT analysis (see Supplementary Table S1).

### IRT analysis: Tasks discriminating cognitive impairment levels

A scree plot showed that the data sufficed the unidimensional assumption that IRT requires (see Figure S1 published as supplementary material online attached to the electronic version of this paper at https://www.cambridge.org/core/journals/european-psychiatry). Item discrimination parameters were computed to map the ability of an item to discriminate latent symptom severity levels (see Table 2 alpha values). For example, word recall had the highest ability to discriminate underlying cognitive impairment levels (α=1.92). Four ADAS-Cog tasks (spoken language ability, comprehension of spoken language, remembering test instruction, and word finding difficulty) had low item discrimination parameters (i.e., these tasks lacked the ability to discriminate underlying cognitive impairment levels). Hence, the aforementioned four tasks were considered inappropriate for the IRT-based short-scale, leaving seven possible ADAS-Cog tasks (word recall, commands, naming, constructional praxis, ideational praxis, orientation, word recognition).

**Table 2.** Item parameters from IRT

Item	α	β_1_	β_2_	β_3_	β_4_	β5	β6	β7	β8	β9	β10	β11	β12
Word recall	1.92*****	−4.82	−4.45	−4.08	−3.33	−2.46	−1.55	−0.66	0.19	1.21	2.59*		
Commands	1.32***	−0.06	1.02	1.84	3.34*	4.63*							
Naming	1.28***	−0.17	−0.09	−0.06	1.51	2.68*							
Constructional praxis	0.96***	−1.55	0.95	2.00*	3.98*	5.93*							
Ideational praxis	1.12***	0.01	1.36	2.09*	2.63*	3.34*							
Orientation	1.41****	−2.26	−1.33	−0.66	−0.07	0.62	1.3	2.35*	4.45*				
Word recognition	1.25***	−4.41	−3.17	−2.25	−1.56	−1.08	−0.58	−0.14	0.3	0.76	1.22	1.74	2.49*
Spoken language ability	−0.59*	3.80*	2.82*	2.55*	2.51*	−2.47							
Comprehension of spoken language	−0.62*	3.08*	2.22*	2.13*	2.11*	−2.02							
Remembering test instruction	−0.76*	2.84*	1.86	1.51	0.71	−2.18							
Word finding difficulty	−0.66*	2.57*	1.69	1.35	1.32	−1.25							

*Note:* Item discrimination parameters (α) map the ability of an item to discriminate latent cognitive impairment levels. Discrimination parameter values (α) that range from 0.01 to 0.24 are very low, 0.25 to 0.64 low, 0.65 to 1.34 moderate, 1.35 to 1.69 high, and over 1.7 are very high (Baker, 2001). βs are standardized estimates of the 0.5 probability of endorsing a higher cognitive impairment rating where negative values indicate progression to the next response is unlikely.

### IRT analysis: ADAS-cog information ascertained at different cognitive impairment levels

**Figure 1.**
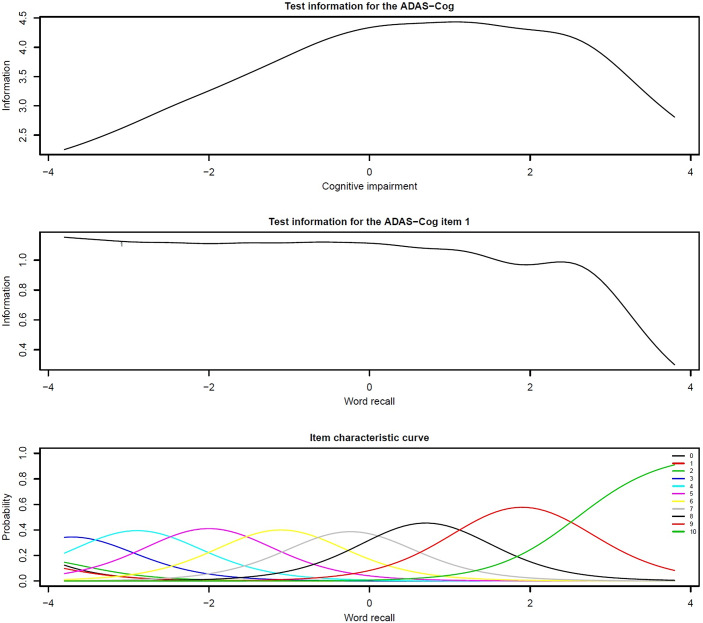
Item response figures. Note: The horizontal axis denotes the underlying latent trait of cognitive impairment.

Task information (reliability) is ascertained by IRT for the total scale and each task. The topmost plot in Figure 1 shows the test information along the vertical axis at different cognitive impairment levels along the horizontal axis for the ADAS-Cog total. Figure 1 (top panel) suggests that the ADAS-Cog is more reliable at moderate and moderately high impairment levels but displays a reliability that is not satisfactory at low and very high cognitive impairment levels. Figure 1 (middle panel) shows that the information ascertained by word recall is moderate across impairment levels up to severe levels of impairment from which the information ascertained is low.

Of the remaining seven possible ADAS-Cog tasks, the amount of information captured ranged from low to moderate. Word recall captured information at moderate cognitive impairment levels, commands from moderate to high levels, naming at moderate levels, constructional praxis from low to high levels, ideational praxis from moderate to high levels, orientation from moderate to high levels, and word recognition from very low to high levels (for information plots for all tasks, see Figures S2 and S3 published as supplementary material online attached to the electronic version of this paper at https://www.cambridge.org/core/journals/european-psychiatry).

### IRT analysis: Response options

Based on item characteristic curves and the probability of a response option being endorsed (Table 2 beta values), we aimed to remove overlapping response options. For instance, the bottom panel of Figure 1 shows that response option 10 is endorsed with a high likelihood at higher impairment levels. All seven possible ADAS-Cog tasks had at least one response option that would likely be required (see Figure S5 published as supplementary material online attached to the electronic version of this paper at https://www.cambridge.org/core/journals/european-psychiatry and Table 2 beta values). However, not all response options appeared to be necessary.

We examined Table 2 (and see Figure S5 published as supplementary material online attached to the electronic version of this paper at https://www.cambridge.org/core/journals/european-psychiatry) to identify and remove superfluous response options. We identified superfluous sources of information for each of the items: word recall (9–10 errors captured severe impairment, and the remaining response options appeared not to capture severe impairment); commands (up to 3 commands incorrect did not appear to have differential utility in capturing impairment, and subsequent commands incorrect slightly superfluous); naming (the options did not capture severe cognitive impairment except five: “9–11 items incorrect”); constructional praxis and ideational praxis (options 0–3 were unlikely to result in a subsequent rating, and 4 and 5 overlapped to moderate to severe capture impairment); orientation (response options 6–8 reflected more severe impairment); and word recognition (12 incorrect responses represented severe impairment, otherwise transition was unlikely and the item responses were quite superfluous).

### The ADAS-Cog IRT short-scale scoring key

Based on the above, we recoded the IRT-based ADAS-Cog short version as follows: word recall (0 except 9–10 recoded as 1); commands (up to 3 as 0, otherwise 1); naming (0 except five as 1); constructional praxis and ideational praxis (options 0–3 as 0, and 4 and 5 as 1); orientation (0–5 as 0, 6–8 as 1); and word recognition (0 except 12 as 1). For consistency and ease of future use, dichotomous scoring was implemented.

### Mixed models

**Figure 2.**
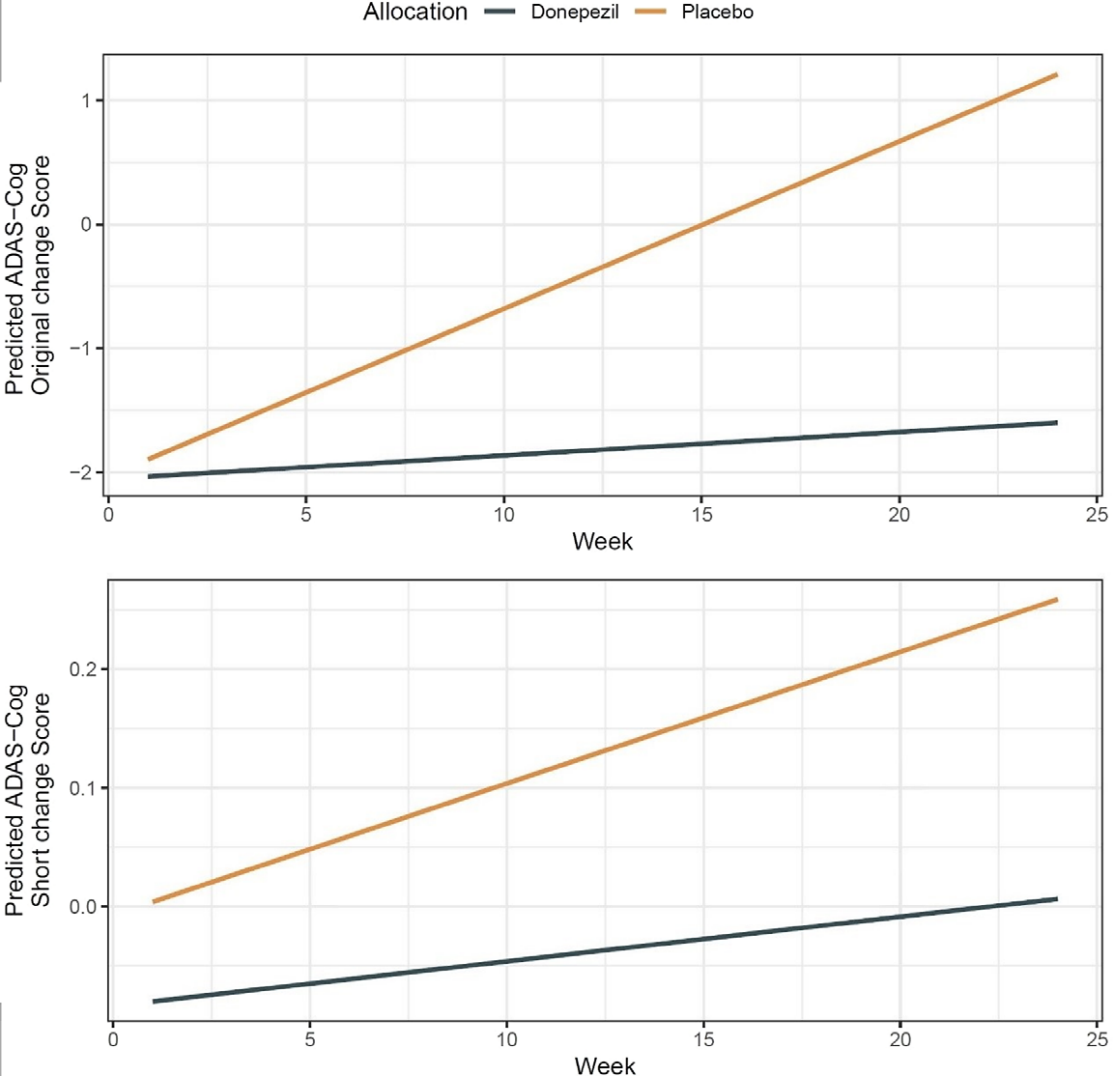
Mixed model modeling changes in the original and short *Alzheimer*’*s Disease Assessment Scale Cognitive Subscale* (ADAS-Cog) up to 24 weeks. Note: Upper figure is the original ADAS-Cog and the lower is the short ADAS-Cog based item response theory.

The bivariate correlation at baseline, at week 12, and week 24 of the short and original ADAS-Cog measures was 0.7 across time points. MRMMs were implemented to contrast the original and IRT-based short ADAS-Cog (Figure 2). The marginal means differed between the original and short ADAS-Cog (original version: donepezil = −1.85, 95% CI = −2.16, −1.53, placebo = −0.38, 95% CI = −0.77, −0.00; short version: donepezil = −0.04, 95% CI = −0.10, −0.02, placebo = 0.11, 95% CI = 0.05, 0.18) were smaller for donepezil than placebo. Based on the marginal means, examination of the effect sizes showed that placebo and donepezil separated more for the original than the short ADAS-Cog version, but the bootstrapped confidence intervals overlapped between versions (ADAS-Cog original ES = 0.33, 95% CI = 0.29, 0.40, ADAS-Cog short ES = 0.25, 95% CI = 0.23, 0.34).

## Discussion

Based on five pivotal clinical trials of donepezil compared with placebo for Alzheimer’s disease (N = 2,198), we implemented IRT to shorten the ADAS-Cog and examined whether this short version could separate treatment and placebo groups in a manner similar to the original version. We identified a short ADAS-Cog that consisted of seven items and found that it separated placebo from donepezil in these trials.

IRT identified a short ADAS-Cog consisting of 7 items with dichotomous response options, in contrast to the original, which consists of 11 items with multiple response options. In our estimation, assuming the ADAS-Cog takes 30 min to administer, the test-time for the short version may be approximately 18 min or less, because the short version has seven items (36.37% fewer items than the original ADAS-Cog) and two response options (to ease future administration).

Based on mixed modeling, scores on the ADAS-Cog change short version were separated between placebo from donepezil in these individual participant trial data. Also, mixed modeling to examine ADAS-Cog change showed conclusions concerning efficacy were similar for both the short and original ADAS-Cog scales (i.e., both showed superior efficacy of donepezil compared to placebo). The effect size, however, slightly favored the original compared to the short scale.

### Limitations and conclusions

Our study has several primary strengths, such as the use of individual-level participant data. Nonetheless, our study has notable limitations. First, clinical trial selection criteria restrict generalizations from clinical trial data to the general population [26, 27]. Hence, caution is warranted regarding generalizing from the current results to clinical treatment settings. To inform clinical practice, replicating the results in large-scale naturalistic studies with extended observation periods may be warranted. Second, unmeasured factors (e.g., delusions) may have confounded the study results. Nonetheless, the data common to all the trials did not contain such other information. Hence, our study may suffer from residual confounding, and future research may wish to account for other potential confounders. Third, our results are restricted to donepezil and placebo. Research is warranted to scrutinize the generalizability of these results to other antidementia drugs. Fourth, the study duration was restricted to 24 weeks of follow-up. Given the course of cognitive decline in Alzheimer’s disease, further research is warranted with longer study durations. Fifth, an independent prospective study is warranted to test the validity of the scale.

The clinical trials in our study were completed over a decade ago. Today, a significant proportion of participants would not receive a research diagnosis of Alzheimer’s disease. Specifically, perhaps up to 30% would receive diagnoses for other neurodegenerative disorders, including vascular or mixed dementia, based on current-day research diagnostic criteria that involve biomarkers, such as amyloid PET, to confirm neuropathology in Alzheimer’s disease according to the 2018 NIA-AA Research Framework [28]. However, the use of biomarkers is yet to translate to daily clinical practice [29]. In current daily clinical practice, the symptomatological diagnostic criteria, including DSM-5 [30] and NINCDS-ADRDA [31], are the basis for the prescription of donepezil and other antidementia drugs, as were done in the trials included in the current study.

Among the strengths of the current study design are the amount of evidence (five pivotal clinical trials) and the relatively large sample, which make the results robust. These features reinforce our faith in the robustness of the analysis. Clinically, a short ADAS-Cog with a strong correlation with the original offers possibilities in reducing the trial participant burden while keeping reliability intact. In sum, the current study contributes to knowledge on Alzheimer’s disease by identifying a short version of the ADAS-Cog with potential use for treatment monitoring in moderate-stage Alzheimer’s disease.

## Supporting information

Levine et al. supplementary material Levine et al. supplementary material

## Supplementary material

The supplementary material for this article can be found at http://doi.org/10.1192/j.eurpsy.2024.14.
