# Supplementary material for: Shortening the Alzheimer’s disease assessment scale cognitive subscale
Source: Eur Psychiatry. 2024 Feb 23;67(1):e19. doi: 10.1192/j.eurpsy.2024.14 (PMC10966609; doi:10.1192/j.eurpsy.2024.14)

Supplement to

Levine et al. Shortening the Alzheimer's Disease Assessment Scale Cognitive Subscale

Figure S1: Screeplot 2

Figure S2: Test Information curve of the ADAS-Cog total scale 3

Figure S3: All Item Information Curves Superimposed as a Single Plot 4

Figure S4: Item Information Curves 5

Figure S5: Item Characteristic Curves 11

# Figure S1: Screeplot


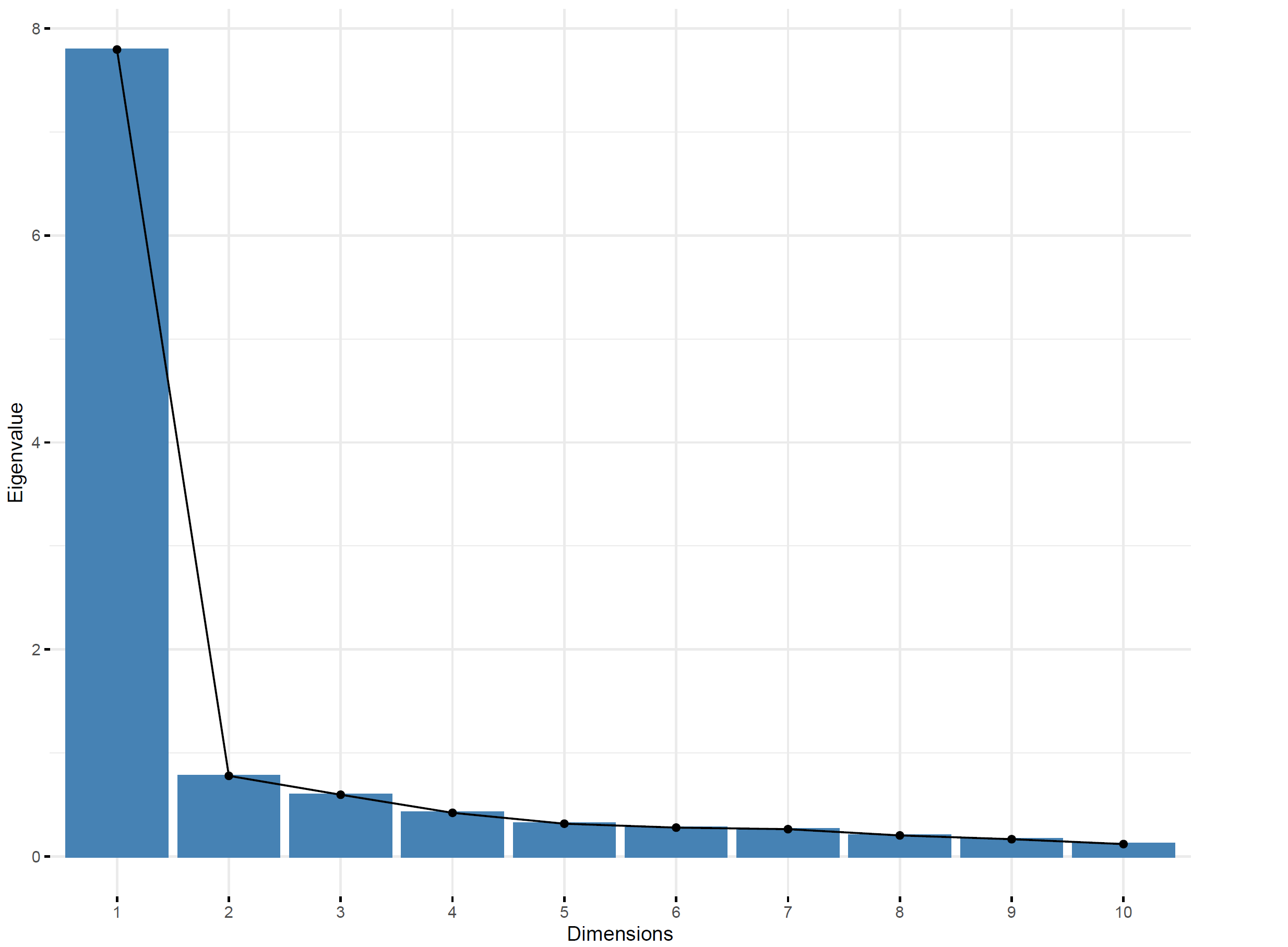


Note. The first eigenvalue was 7.80 and accounted for 70.86% of the variance, and the second eigenvalue was 0.55.

# Figure S2: Test Information curve of the ADAS-Cog total scale


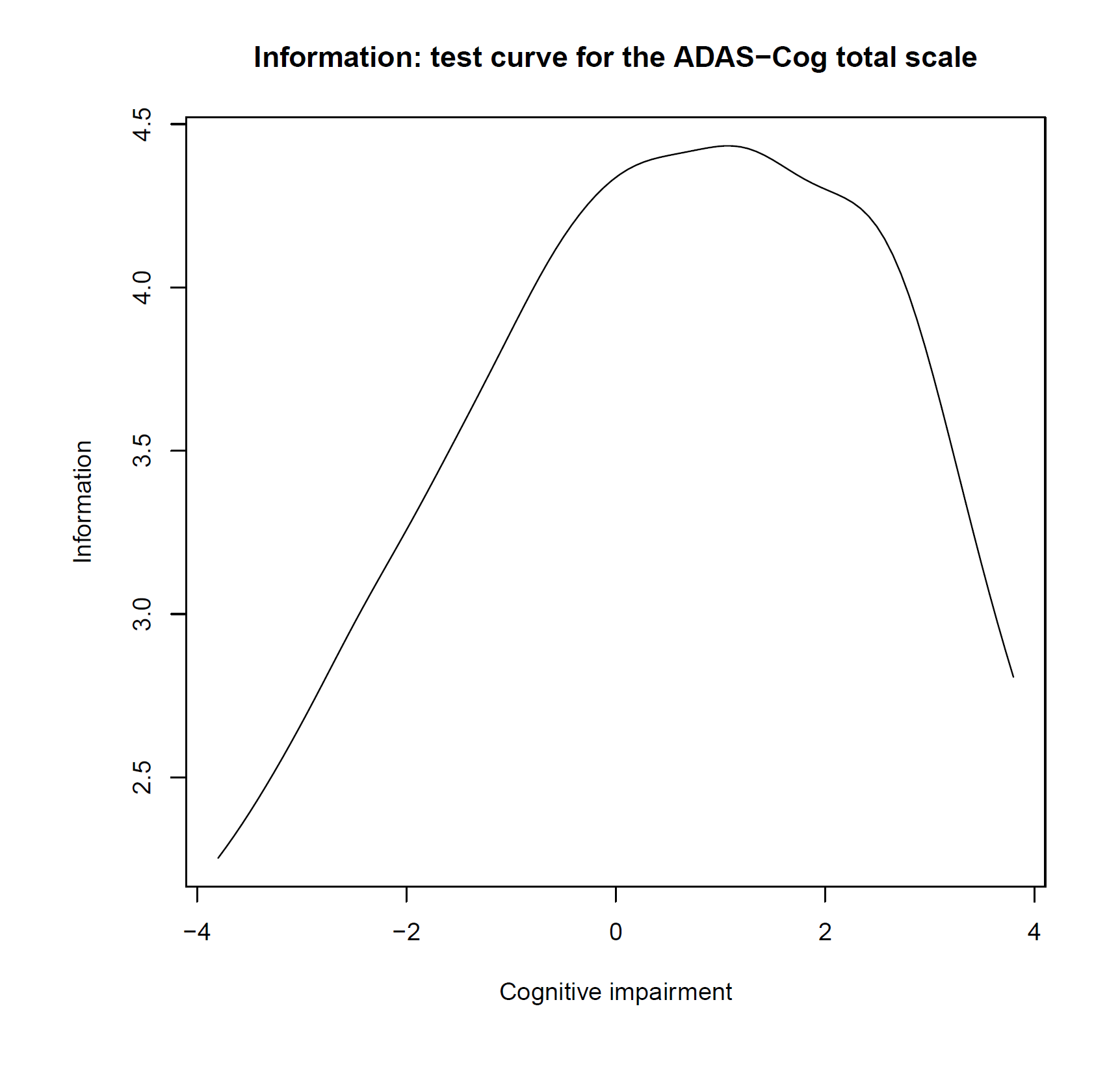


Note. A common rule of thumb is that 0.70 to 0.90 reliability values align with information values ranging from 3.3 to 10, respectively.

# Figure S3: All Item Information Curves Superimposed as a Single Plot


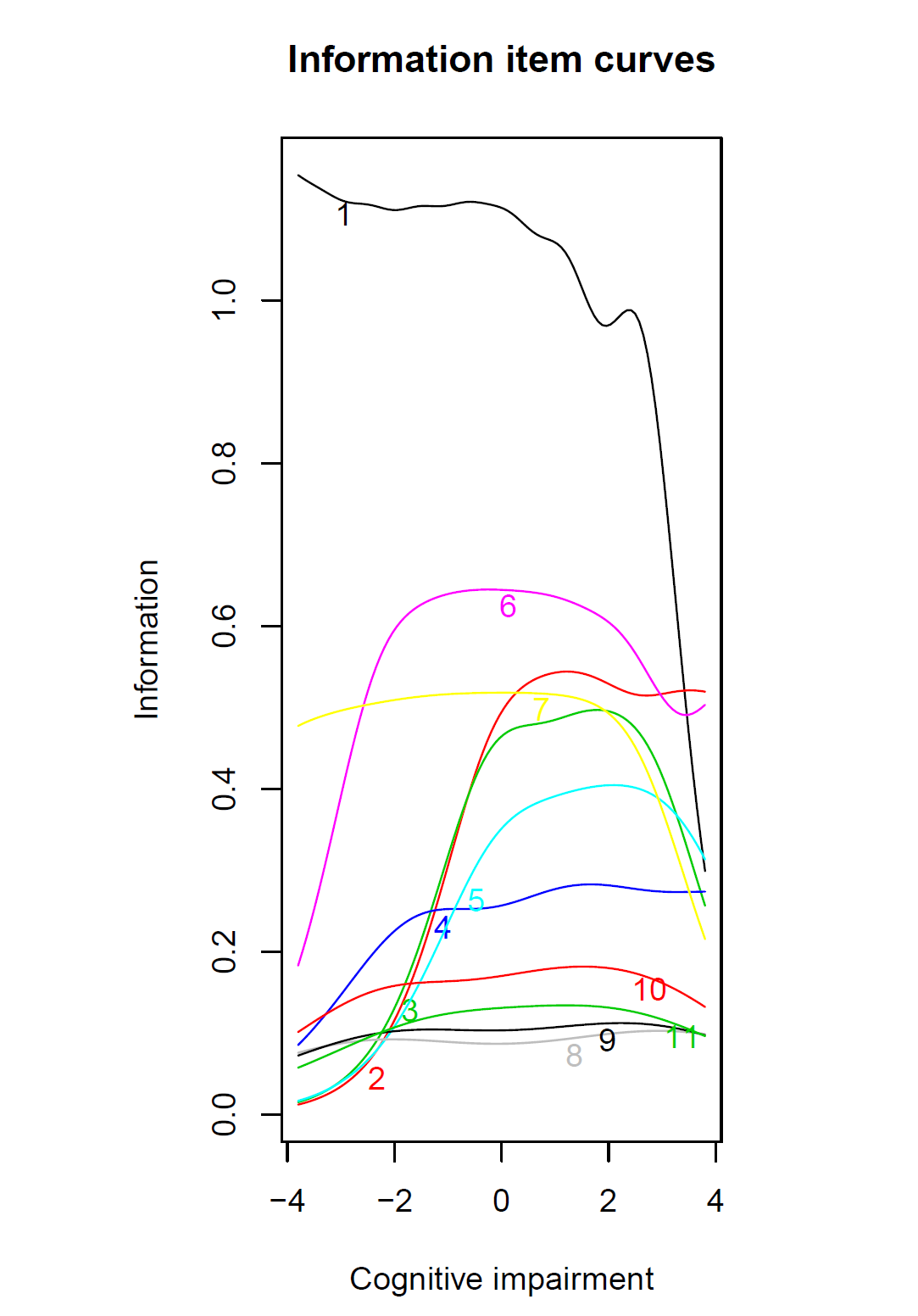


# Figure S4: Item Information Curves


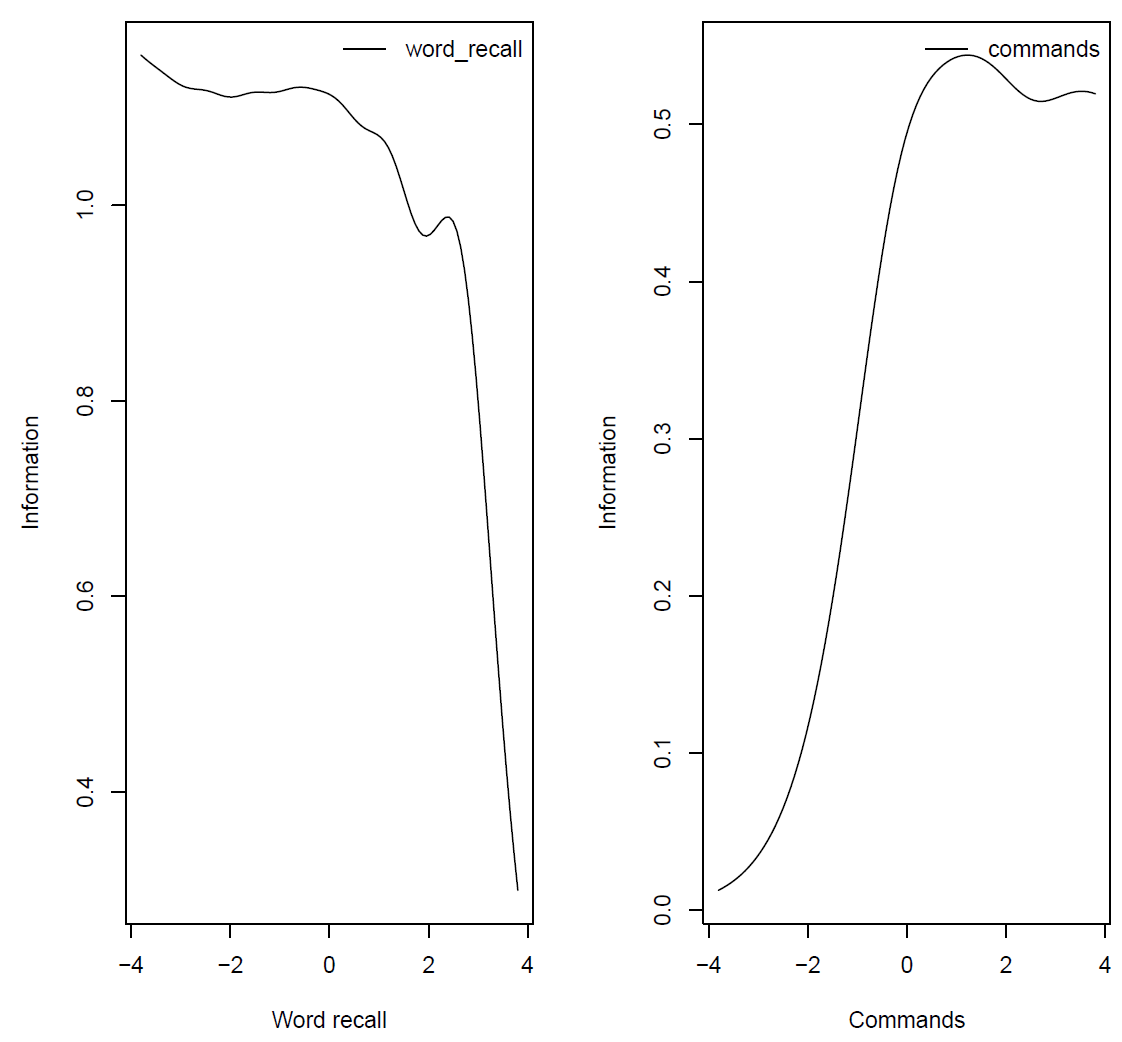


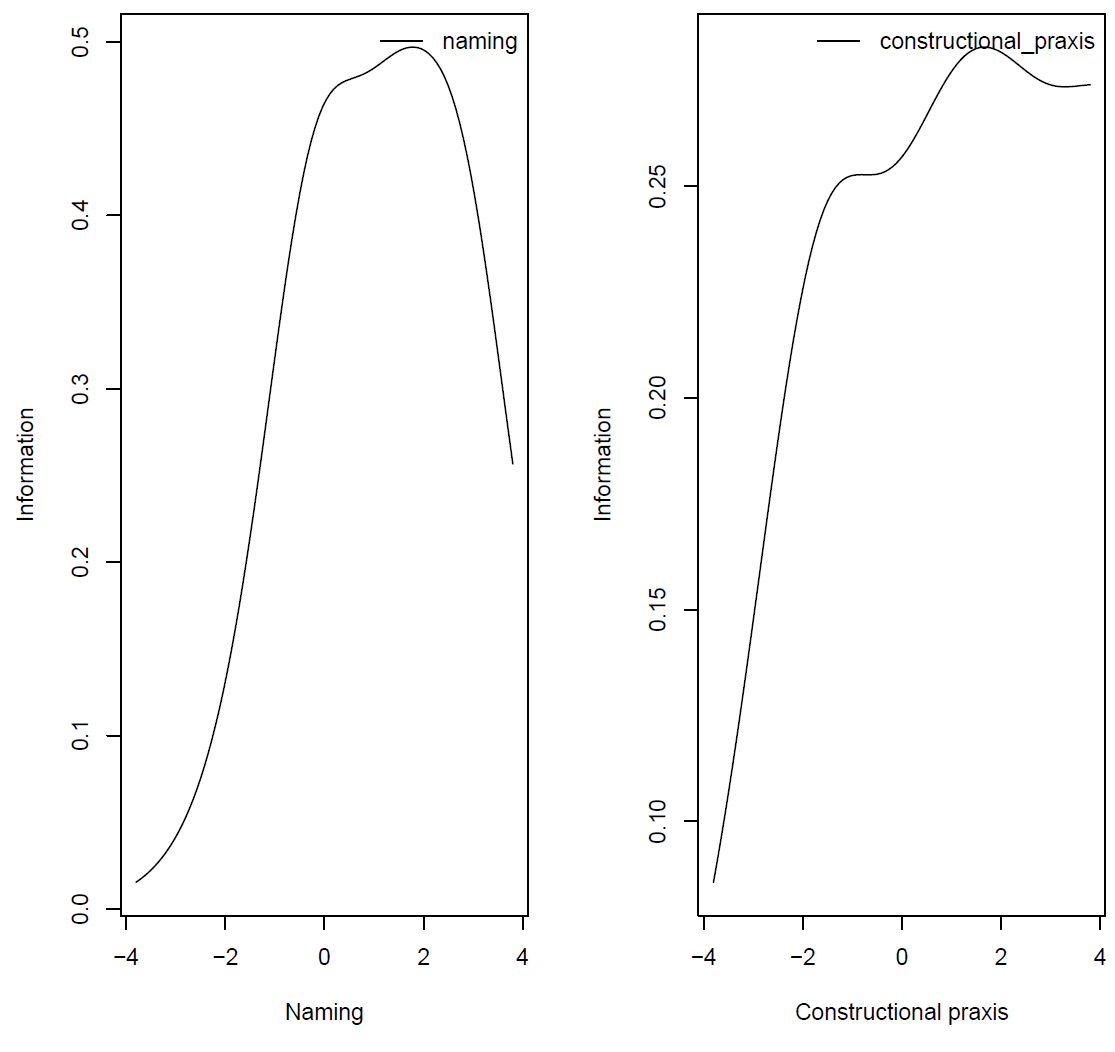


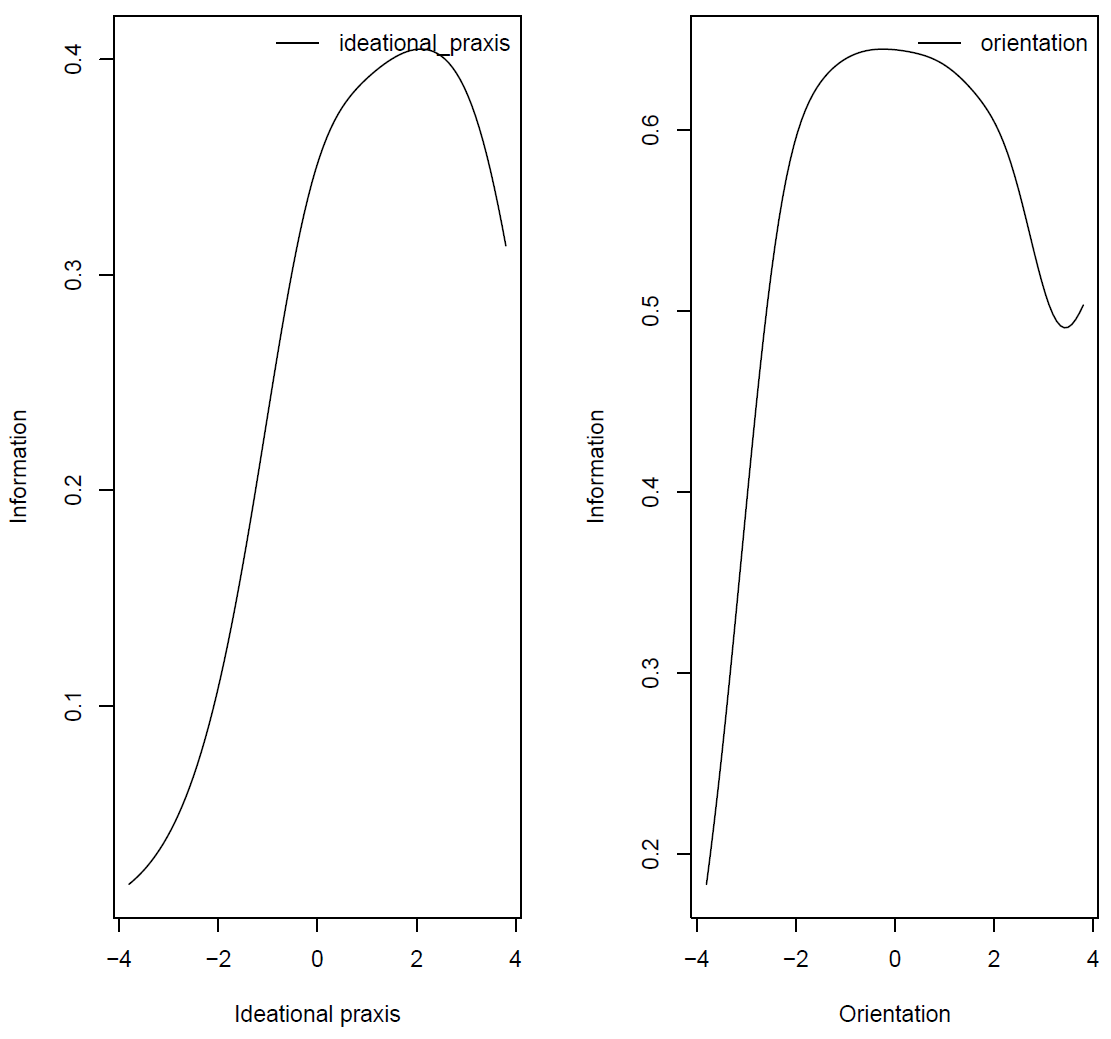


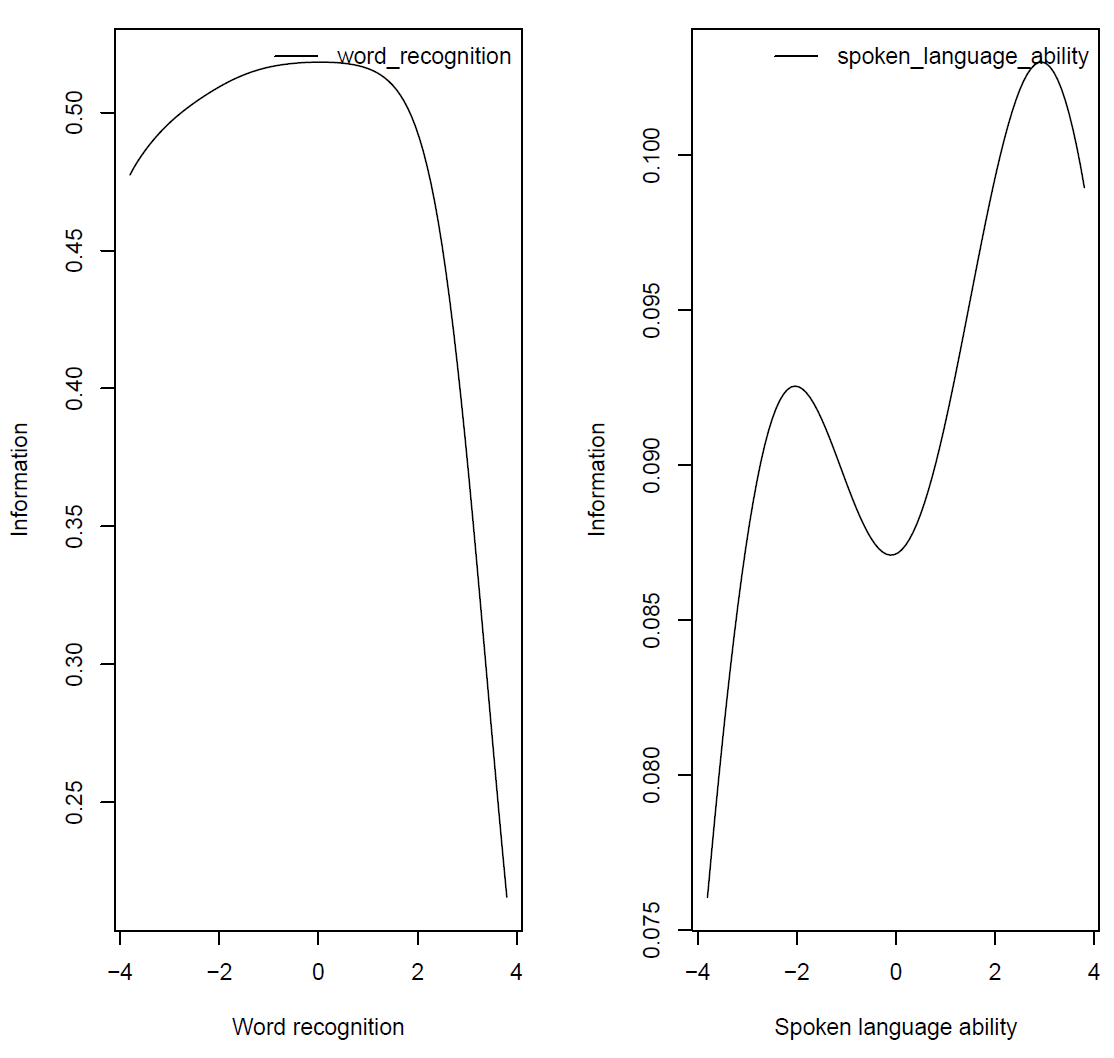


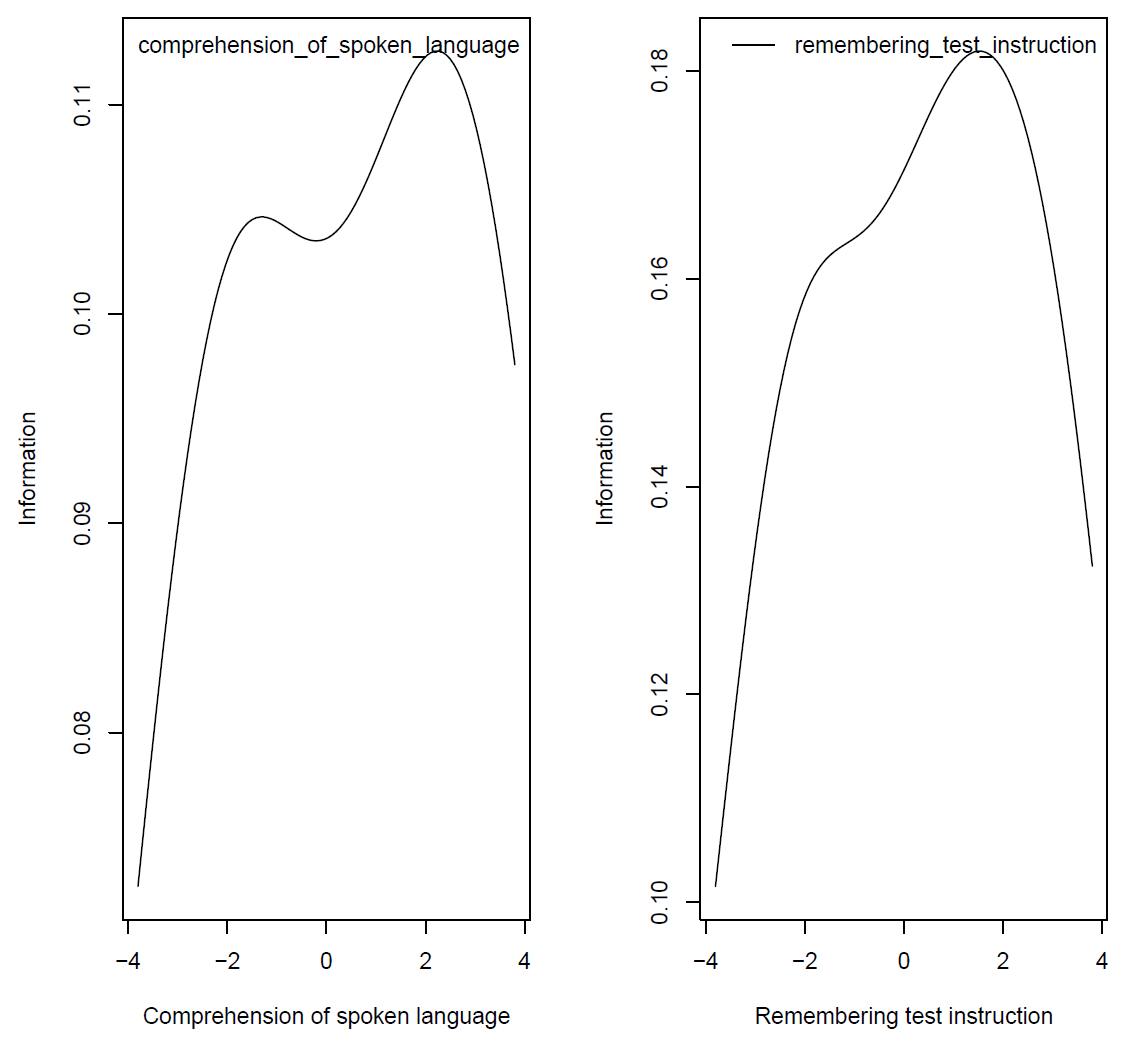


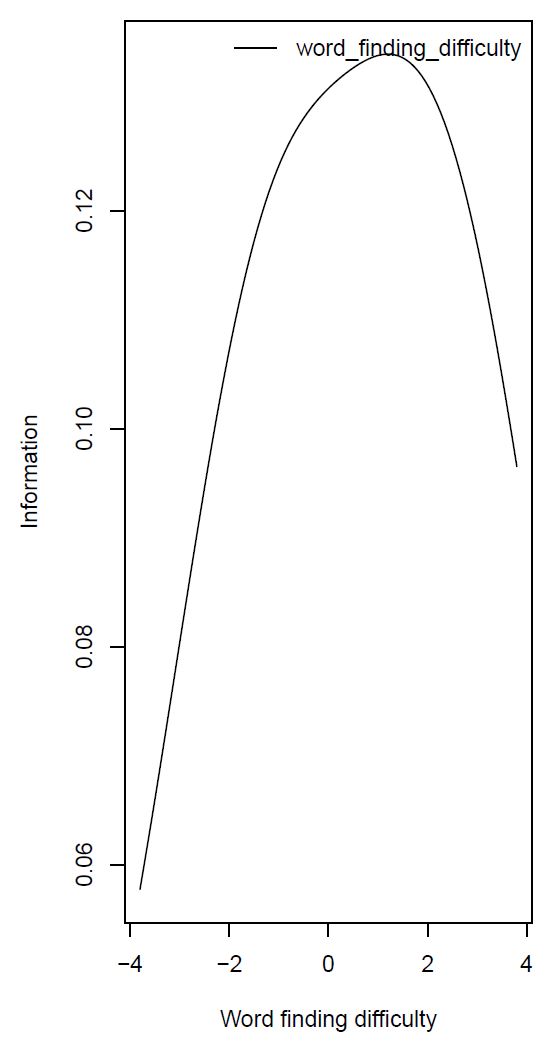


# Figure S5: Item Characteristic Curves


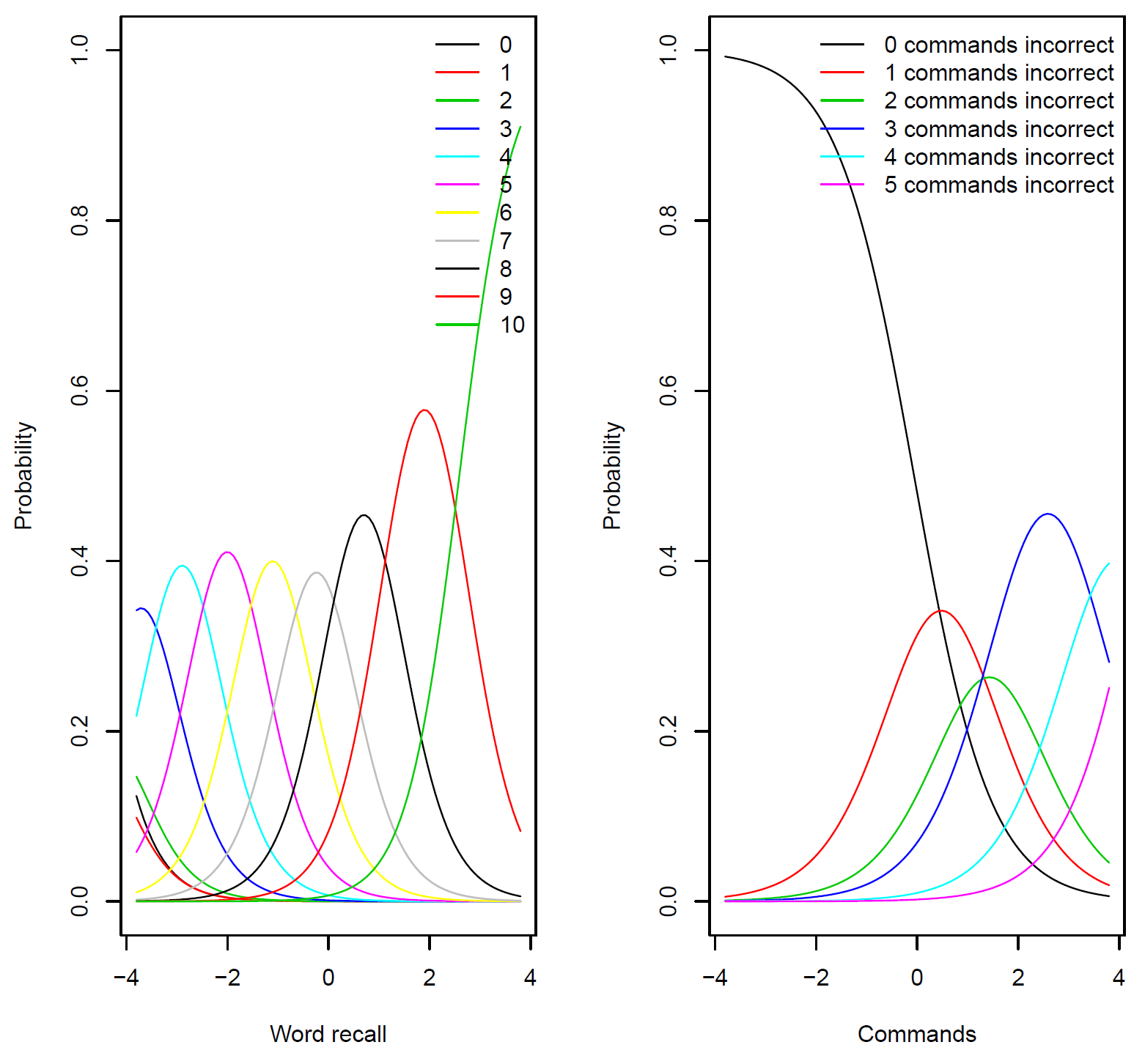


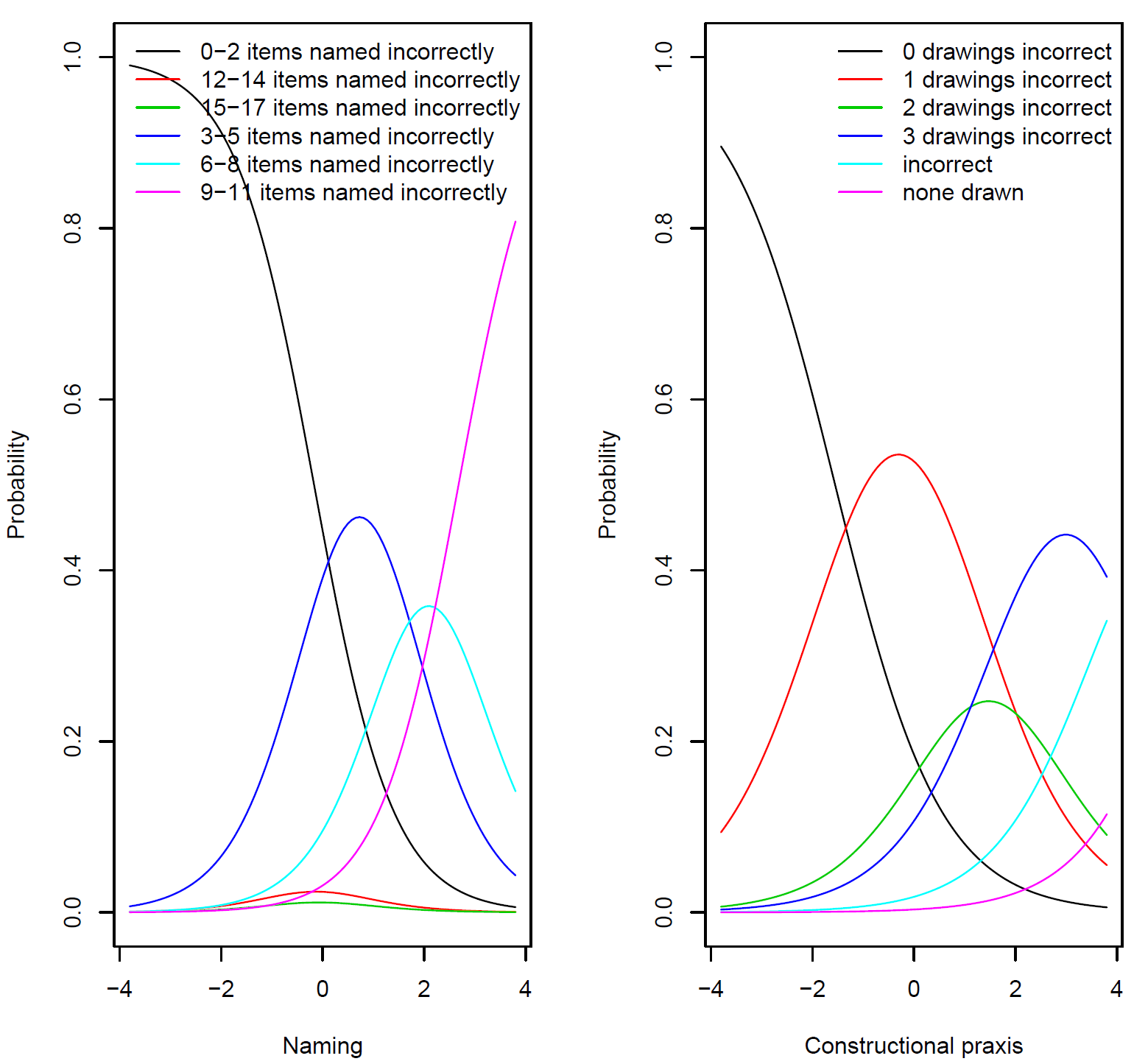


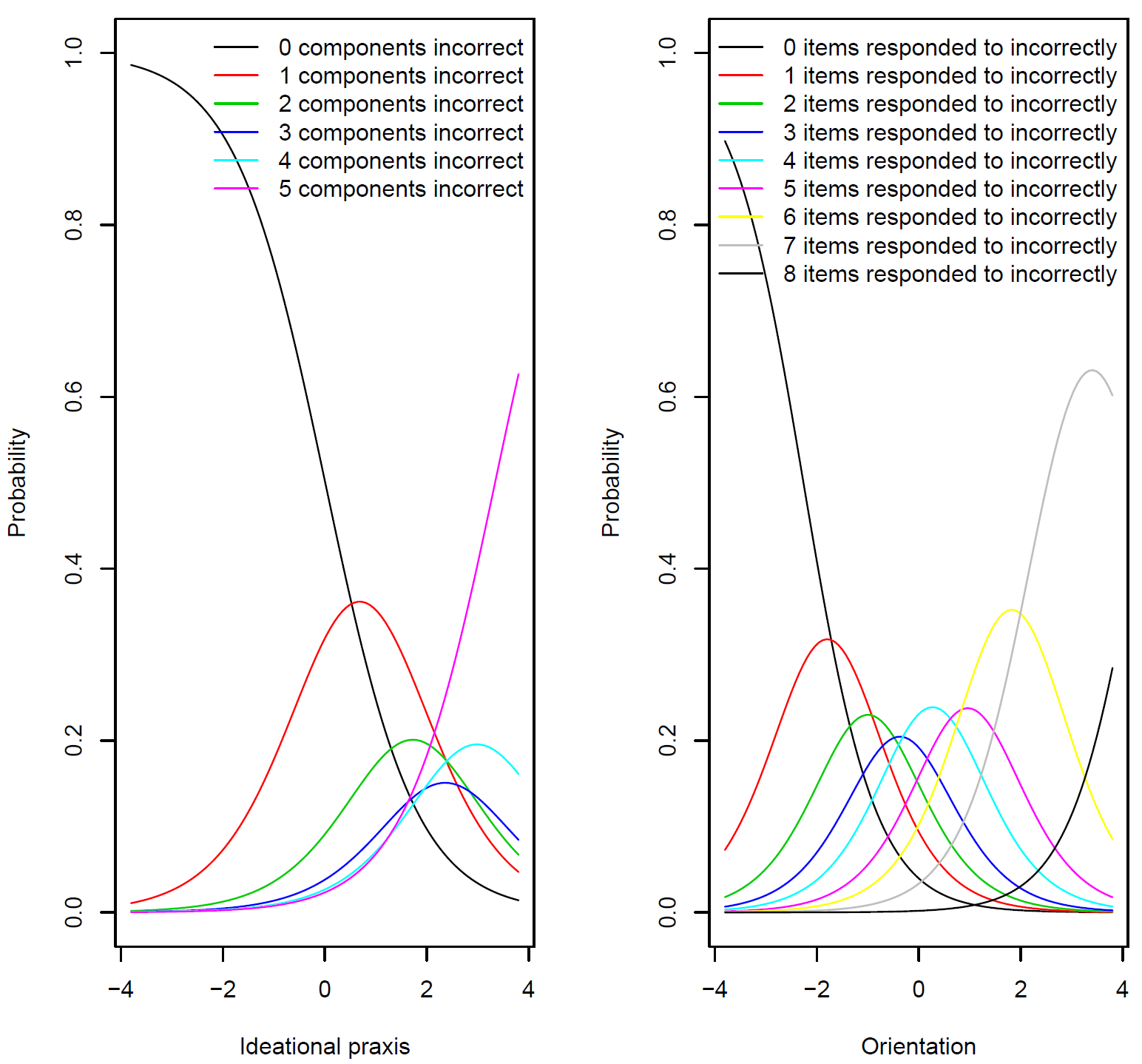


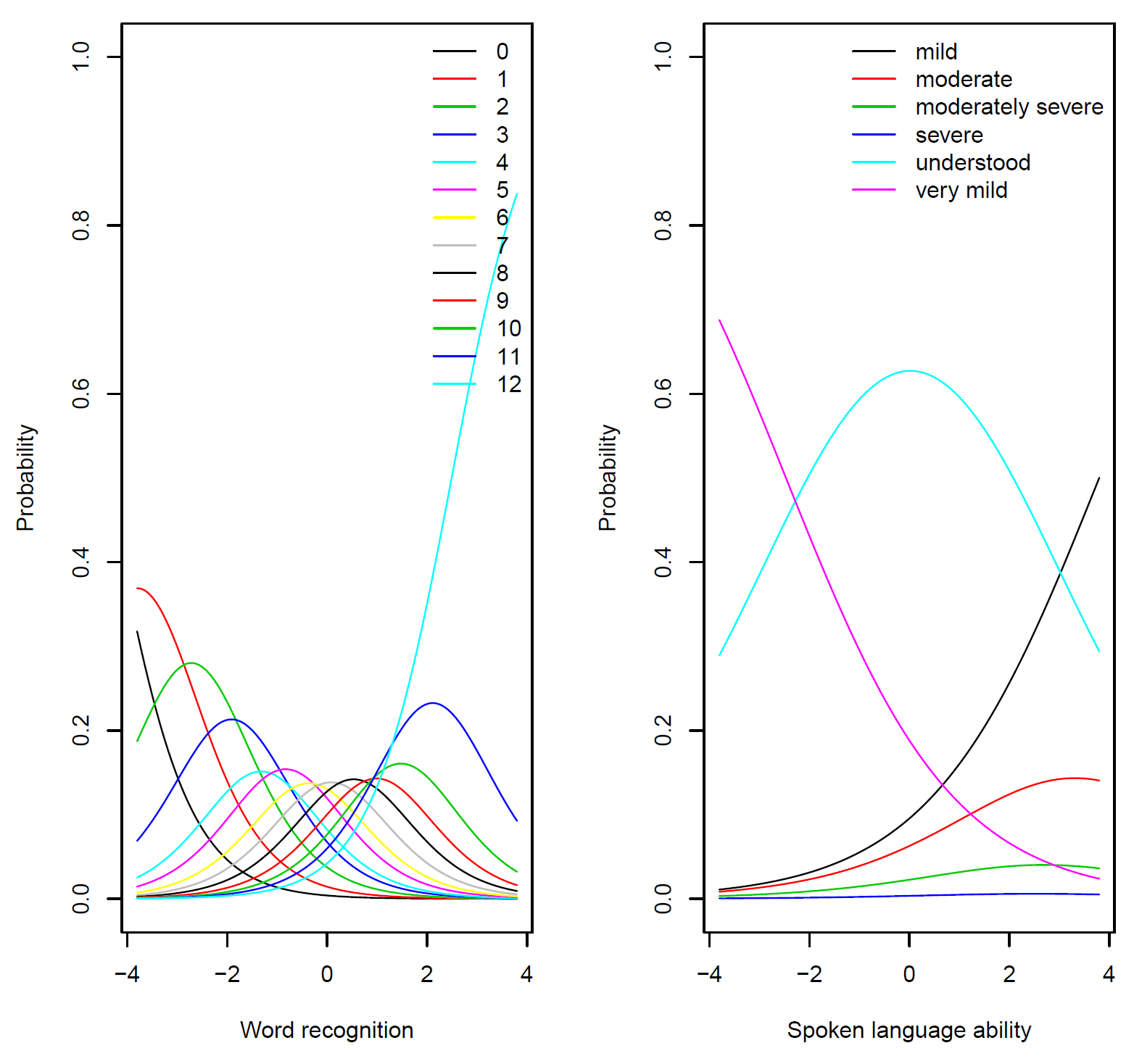


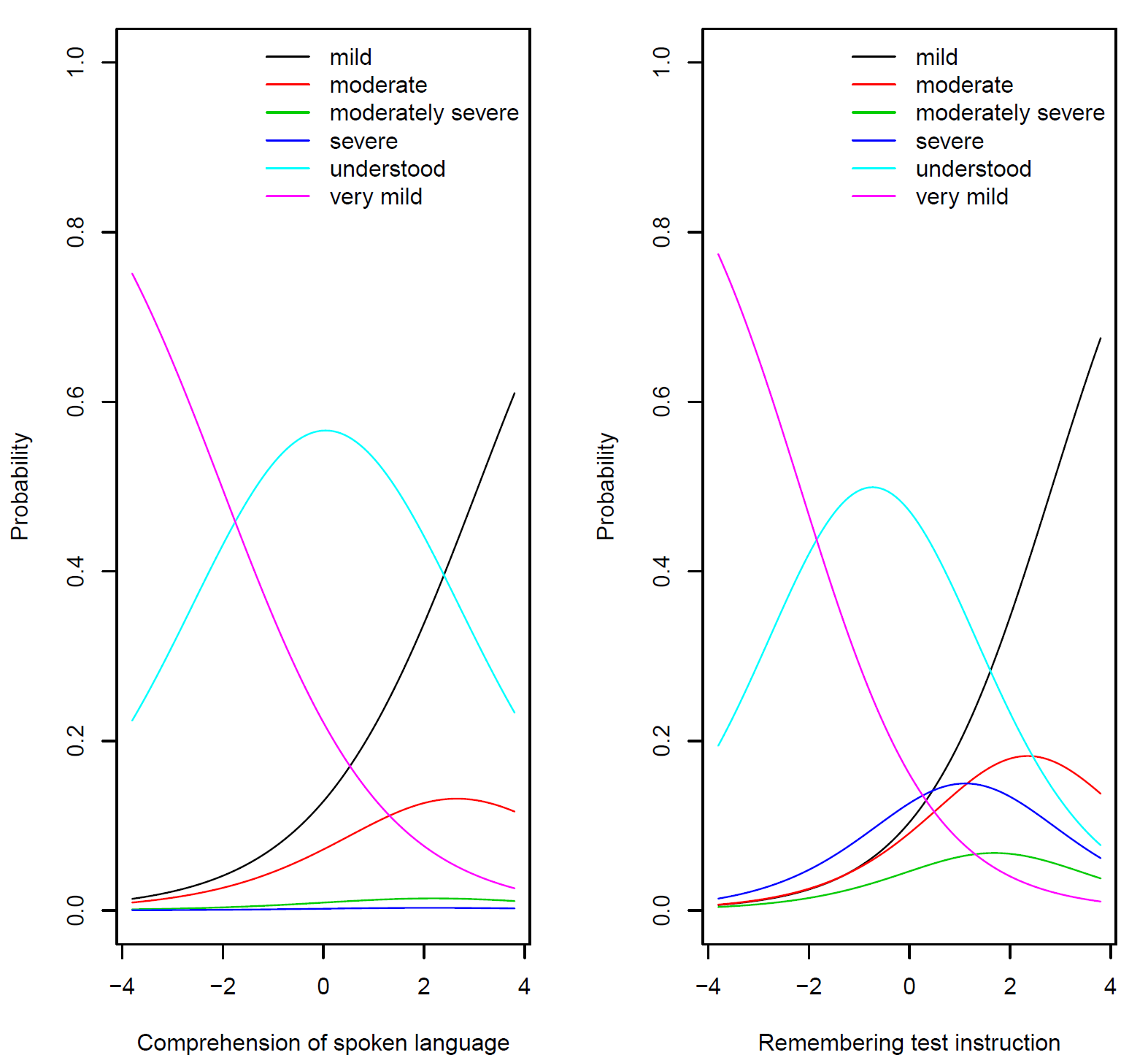


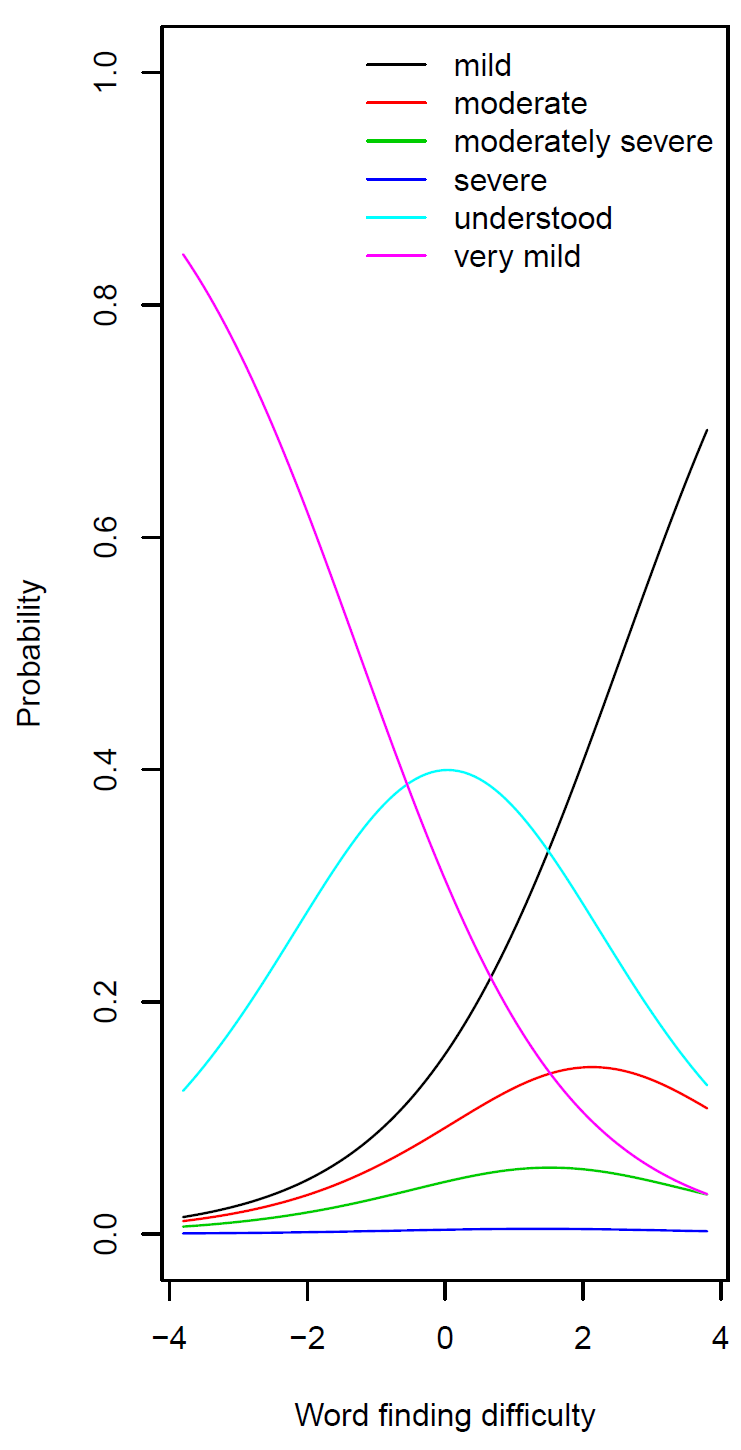

Supplement: Levine et al. supplementary material [file S0924933824000142sup001.docx]
